# *Acinetobacter* spp. Infections in Malaysia: A Review of Antimicrobial Resistance Trends, Mechanisms and Epidemiology

**DOI:** 10.3389/fmicb.2017.02479

**Published:** 2017-12-12

**Authors:** Farahiyah Mohd. Rani, Nor Iza A. Rahman, Salwani Ismail, Ahmed Ghazi Alattraqchi, David W. Cleary, Stuart C. Clarke, Chew Chieng Yeo

**Affiliations:** ^1^Faculty of Medicine, Universiti Sultan Zainal Abidin, Kuala Terengganu, Malaysia; ^2^Faculty of Medicine and Institute for Life Sciences, University of Southampton, Southampton, United Kingdom; ^3^NIHR Southampton Biomedical Research Centre, University of Southampton, Southampton, United Kingdom; ^4^Global Health Research Institute, University of Southampton, Southampton, United Kingdom; ^5^International Medical University, Kuala Lumpur, Malaysia

**Keywords:** *Acinetobacter*, antimicrobial resistance, Malaysia, surveillance data, epidemiology, resistance mechanisms

## Abstract

*Acinetobacter* spp. are important nosocomial pathogens, in particular the *Acinetobacter baumannii*-*calcoaceticus* complex, which have become a global public health threat due to increasing resistance to carbapenems and almost all other antimicrobial compounds. High rates of resistance have been reported among countries in Southeast Asia, including Malaysia. In this review, we examine the antimicrobial resistance profiles of *Acinetobacter* spp. hospital isolates from Malaysia over a period of nearly three decades (1987–2016) with data obtained from various peer-reviewed publications as well as the Malaysian National Surveillance on Antibiotic Resistance (NSAR). NSAR data indicated that for most antimicrobial compounds, including carbapenems, the peak resistance rates were reached around 2008–2009 and thereafter, rates have remained fairly constant (e.g., 50–60% for carbapenems). Individual reports from various hospitals in Peninsular Malaysia do not always reflect the nationwide resistance rates and often showed higher rates of resistance. We also reviewed the epidemiology and mechanisms of resistance that have been investigated in Malaysian *Acinetobacter* spp. isolates, particularly carbapenem resistance and found that *bla*_OXA-23_ is the most prevalent acquired carbapenemase-encoding gene. From the very few published reports and whole genome sequences that are available, most of the *Acinetobacter* spp. isolates from Malaysia belonged to the Global Clone 2 (GC2) CC92 group with ST195 being the predominant sequence type. The quality of data and analysis in the national surveillance reports could be improved and more molecular epidemiology and genomics studies need to be carried out for further in-depth understanding of Malaysian *Acinetobacter* spp. isolates.

## Introduction

*Acinetobacter* spp. are Gram-negative opportunistic pathogens associated with severe nosocomial infections including pneumonia, bloodstream, urinary tract and wound infections, as well as meningitis. The majority of infections are due to the *A. baumannii–A. calcoaceticus* (*Abc*) complex with *A. baumannii* being the most clinically important species (Dijkshoorn et al., 2007; Clark et al., 2016; Gonzalez-Villoria and Valverde-Garduno, 2016). The genus *Acinetobacter* is taxonomically complex with unambiguous identification at the species level particularly problematic (Gundi et al., 2009). *A. baumannii, A. nosocomialis, A. pittii* and *A. calcoaceticus*, which is usually an environmental species, along with two novel pathogenic species, *A. seifertii* and *A. djikshoorniae* cannot be reliably differentiated by phenotypic tests, and are thus usually grouped together as the *Abc* complex (Gerner-Smidt et al., 1991; Nemec et al., 2015; Cosgaya et al., 2016; Marí-Almirall et al., 2017). Accurate identification at the species level requires sequencing of the RNA polymerase β-subunit gene, *rpoB*, and/or the DNA gyrase B gene, *gyrB* (Gundi et al., 2009), with full-length 16S rRNA gene sequencing proven unreliable (Wang et al., 2014).

Carbapenems are broad-spectrum β-lactam antibiotics that have been the treatment of choice for *Acinetobacter* infections, particularly in critically ill patients (Fishbain and Peleg, 2010). However, the increasing prevalence of carbapenem-resistant *A. baumannii*, particularly in the last two decades, has been of immense concern such that carbapenem-resistant *A. baumannii* is now listed as the top priority pathogen in urgent need of new antimicrobials by the World Health Organization in February 2017 (World Health Organization, 2017). This is due to *Acinetobacter* spp., especially *A. baumannii*, having extensive intrinsic antimicrobial resistance mechanisms coupled with the inherent ability to easily acquire new resistance determinants through mobile genetic elements such as plasmids, transposons and genomic islands (Peleg et al., 2008; Doi et al., 2015). Carbapenem-resistant *A. baumannii* is the most common pathogen associated with nosocomial infections in Southeast Asia (Mendes et al., 2013; Suwantarat and Carroll, 2016), a region which groups together 11 nations with disparate incomes and levels of development. The surveillance of antimicrobial resistance among common pathogens was one of the important recommendations issued by the World Health Organization (WHO) in 2001 to slow down the emergence and contain the spread of bacterial resistance (WHO, 2001). Only four Southeast Asian countries, namely Singapore, Thailand, Malaysia and the Philippines have established national antimicrobial surveillance programs; poorer countries such as Myanmar and East Timor (or Timor-Leste) are hampered by limited microbiology laboratory capabilities (Hsu et al., 2017). Malaysia, which is considered as an upper middle income nation and with an active national antimicrobial surveillance program, has surprisingly few publications and little comprehensive data available on *Acinetobacter* spp. infections (McNeil et al., 2016). A recent paper that estimated the mortality attributable to multidrug-resistant pathogens in nosocomial infections in Thailand clearly showed that *Acinetobacter* spp. is the leading cause of hospital-acquired infections with the highest attributable mortality at around 40% (Lim et al., 2016). It would not be surprising if similar burdens of *Acinetobacter* infection are present in neighboring Malaysia but such data have not been published.

In this review, we look at the resistance trends of several antimicrobials for *Acinetobacter* spp. isolated in Malaysia with data obtained from individual studies (which usually involves strains isolated from single institutions/healthcare centers) as well as from the Malaysian National Surveillance on Antibiotic Resistance (NSAR), and spanning a period of nearly three decades, between 1987 and 2016. We also cover the various mechanisms of resistance that have been elucidated, in particular carbapenem resistance, and finally, we review the epidemiological and genomic studies of *Acinetobacter* spp. that have been published, thereby giving us an overview of the state of *Acinetobacter* antimicrobial resistance and epidemiology in this Southeast Asian nation.

## Antibiotic Susceptibility Profiles

The Institute for Medical Research (IMR), Malaysia, publishes the NSAR results from 2003 onward (except year 2006) online ^1^ which surveys isolates from various hospitals throughout Malaysia, including Sabah and Sarawak in Borneo. The number of hospitals involved and the sample sizes differ each year but have increased from just 12 hospitals in 2007 to 41 hospitals in 2016. Prior to 2007, the NSAR data only presented the total number of isolates that were analyzed for that particular year (i.e., for 2003–2005) without indicating the source of these isolates. The names of the participating hospitals were only published from 2009 onward. Nevertheless, the data did not indicate the prevaling resistance rates for individual participating hospitals but rather was analyzed as a total cumulative pool of isolates.

**FIGURE 1:**
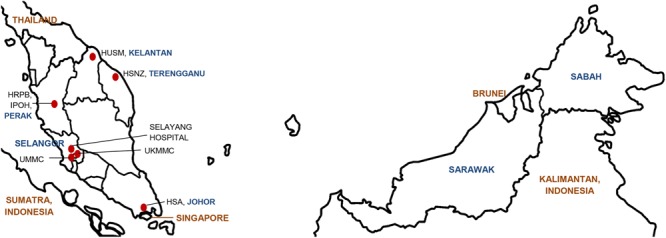
Map of Malaysia indicating the geographical location of the hospitals in which the *Acinetobacter* spp. isolates were obtained for the various individual studies that had been conducted and reviewed in this paper. The various states within Malaysia are indicated in blue whereas neighboring countries are labeled in brown. HUSM, Hospital Universiti Sains Malaysia; HSNZ, Hospital Sultanah Nur Zahirah; HSA, Hospital Sultanah Aminah; HRPB, Hospital Raja Perempuan Bainun; UKMMC, Universiti Kebangsaan Malaysia Medical Centre; UMMC, University Malaya Medical Centre.

The Clinical and Laboratories Standard Institute (CLSI) currently lists 24 antimicrobial agents from nine groups with breakpoints for *Acinetobacter* spp. (CLSI, 2017). A joint initiative between the European Centre for Disease Prevention and Control (ECDC) and the US Centers for Disease Prevention and Control (CDC) led to the development of standard definitions of MDR, extensive drug resistance (XDR) and pandrug resistance (PDR) in an effort to harmonize the antimicrobial resistance surveillance systems (Magiorakos et al., 2012). The ECDC-CDC recommendation for *Acinetobacter* spp. covered 22 of the 24 CLSI antimicrobial agents (omitting piperacillin from the penicillin group and gatifloxacin from the fluroquinolone group; see **Table 1**) (Magiorakos et al., 2012). In the Malaysian NSAR reports, only six groups of antimicrobials were regularly tested (no data was available for antibiotics under the folate pathway inhibitor group and limited data available for the lipopeptides polymyxin B and colistin). The NSAR data do not give any indication on the prevalence of MDR (let alone XDR or PDR) among the isolates that were tested. No mention was made in the NSAR reports to differentiate between infection and colonization and whether the isolates were obtained from hospital-acquired or community-acquired infections. The source of the bacterial isolates (i.e., whether they were isolated from blood, pus, tracheal aspirates, or other clinical samples) were only stated in the NSAR reports of 2015 onward. We are thus unable to assess the quality assurance or the validity of the NSAR data but these are nevertheless presented here as they are the only publically available nationwide data available for Malaysia. Besides NSAR, there were also scattered reports from other researchers throughout Malaysia who obtained *Acinetobacter* spp. samples from various hospitals throughout the country, albeit only in Peninsular Malaysia and not in the states of Sabah and Sarawak in Borneo (see **Figure 1** for the geographical location of these studies). These *Acinetobacter* spp. were isolated from clinical specimens in the respective hospital laboratories and the sources of these isolates were usually presented in these reports. However, whether these were hospital-acquired or community-acquired infections are not known. The panel of antibiotics used by these researchers differs from the NSAR report, thus making meaningful comparisons difficult. Nevertheless, there are some common antimicrobials that were used throughout the few research papers that have been published and here, we summarize and review these results.

**Table 1:** List of antimicrobials recommended by the European Centre for Disease Prevention and Control (ECDC) and the United States Centers for Disease Prevention and Control (CDC) for standard definitions of multidrug resistance, extensive drug resistance and pandrug resistance for *Acinetobacter* spp. (Magiorakos et al., 2012) along with the antimicrobial agents with available breakpoints as given by the Clinical and Laboratories Standard Institute (CLSI) in its 2017 edition (CLSI, 2017).

Antimicrobial agent	Inclusion in ECDC-CDC
with CLSI breakpoints	recommendation
**Penicillins**	
Piperacillin	No
**β-lactam/β-lactamase inhibitor**	
Ampicillin/Sulbactam	Yes
Piperacillin/Tazobactam	Yes
Ticarcillin/Clavulanante	Yes
**Cephams**	
Ceftazidime	Yes
Cefepime	Yes
Cefotaxime	Yes
Ceftriaxone	Yes
**Carbapenems**	
Doripenem	Yes
Imipenem	Yes
Meropenem	Yes
**Lipopeptides**	
Colistin	Yes
Polymyxin B	Yes
**Aminoglycosides**	
Gentamicin	Yes
Tobramyxin	Yes
Amikacin	Yes
Netilmycin	Yes
**Tetracycline**	
Doxycycline	Yes
Minocycline	Yes
Tetracycline	Yes
**Fluoroquinolones**	
Ciprofloxacin	Yes
Levofloxacin	Yes
Gatifloxacin	No
**Folate pathway inhibitors**	
Trimethoprim-sulfamethoxazole	Yes

*Antimicrobial groups are given in bold.*

### Carbapenems

**FIGURE 2:**
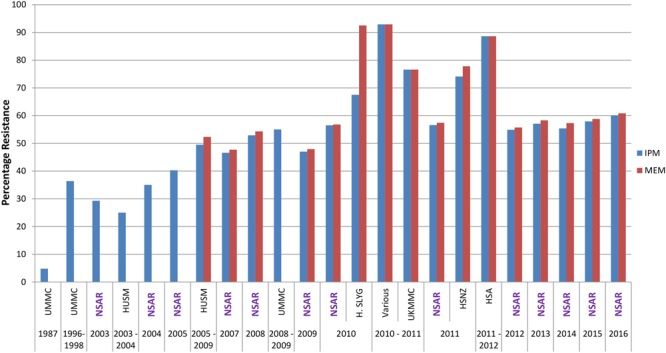
Carbapenem resistance rates for Malaysian *Acinetobacter* spp. isolates (1987–2016). IMP, imipenem; MEM, meropenem. Data from the National Surveillance for Antibiotic Resistance (NSAR) is included and labeled as “NSAR” in purple-colored fonts. Data from the other studies are as follows: UMMC from 1987 and between 1996 and 1998, (Misbah et al., 2004); HUSM between 2003 and 2006, (Deris et al., 2009); and between 2005 and 2009, (Ariffin et al., 2012); UMMC between 2008 and 2009, (Dhabaan et al., 2012); Hospital Selayang (H. SLYG) in 2010, (Nazmul et al., 2012); UKMMC between 2010 and 2011, (Biglari et al., 2015, 2017); Various, collected from various hospitals mainly around the town of Ipoh in the state of Perak in 2010 and 2011, (Kor et al., 2014); HSNZ in 2011, (Lean et al., 2014); and Hospital Sultanah Aminah (HSA) between 2011 and 2012 (Dhanoa et al., 2015).

Carbapenems are usually the drug of choice for serious *Acinetobacter* infections; nevertheless their utility is increasingly compromised by the rapid emergence of resistance (Peleg et al., 2008; Doi et al., 2015). *Acinetobacter* spp. isolates (*n* = 21) from the UMMC, which is located in the capital city of Kuala Lumpur, and collected in 1987 showed imipenem resistance rates of only 4.8% but a decade after that, imipenem resistance rates have increased to 36.4% for isolates collected between 1996 and 1998 (*n* = 88) (**Figure 2**) (Misbah et al., 2004). The first NSAR data in 2003 showed that the national resistance rate for meropenem was slightly below 30% and this was also reflected in a study of isolates from HUSM, located in the northeastern state of Kelantan, from 2003–2004 (Deris et al., 2009). However, by 2008, the NSAR data showed that the resistance rates for meropenem as well as imipenem have reached 50%. Nevertheless, there has not been any drastic increase in the nationwide carbapenem resistance rates from 2008–2016 which has stayed around 50–60%. Several studies on *A. baumannii* isolates from individual hospitals showed carbapenem resistance rates higher than the national average: ICU isolates from the UMMC collected from 2006–2009 showed very high resistance rates for imipenem at 96.5% and meropenem at 98.2% (Kong et al., 2011), as did isolates from several ward in Hospital Selayang (located also in Kuala Lumpur) in 2010 with a 92.5% resistance rate for meropenem whereas the imipenem resistance rate was lower at 67.5% (Nazmul et al., 2012). Likewise, *A. baumannii* isolates collected in 2010 and 2011 from various ward in HSA in the southern state of Johor, displayed resistance rates of 88% for both imipenem and meropenem (Dhanoa et al., 2015). Resistance rates of >70% were also reported for isolates from UKMMC (located south of Kuala Lumpur) in 2010–2011 (Biglari et al., 2015, 2017) and HSNZ (located in the east coast state of Terengganu) in 2011(Lean et al., 2014).

### Cephalosporins

**FIGURE 3:**
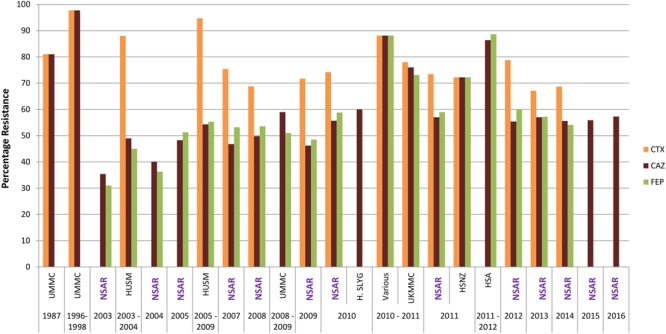
Cepholosporin resistance rates for Malaysian *Acinetobacter* spp. isolates (1987–2016). CTX, cefotaxime; CAZ, ceftazidime; and FEP, cefepime. Data from the National Surveillance for Antibiotic Resistance (NSAR) is included and labeled as “NSAR” in purple-colored fonts. Data from the other studies are as follows: UMMC from 1987 and between 1996 and 1998, (Misbah et al., 2004); HUSM between 2003 and 2006, (Deris et al., 2009); and between 2005 and 2009, (Ariffin et al., 2012); UMMC between 2008 and 2009, (Dhabaan et al., 2012); Hospital Selayang (H. SLYG) in 2010, (Nazmul et al., 2012); UKMMC between 2010 and 2011, (Biglari et al., 2015, 2017); Various, collected from various hospitals mainly around the town of Ipoh in the state of Perak in 2010 and 2011, (Kor et al., 2014); HSNZ in 2011, (Lean et al., 2014); and Hospital Sultanah Aminah (HSA) between 2011 and 2012 (Dhanoa et al., 2015).

The national *A. baumannii* resistance rates for the extended-spectrum cephalosporins of the third generation, ceftazidime, and the fourth generation, cefepime, were around 30% in 2003 but increased to around 50% between 2005 and 2009 (**Figure 3**). The resistance rates for both ceftazidime and cefepime remained within the 50–60% range throughout 2010–2014. From 2015 onward NSAR only reported rates for ceftazidine, which maintained between 55 and 60%. Reports of strains that were isolated from individual hospitals showed higher resistance rates for ceftazidime and cefepime when compared to the national average: strains from HSA in 2010 and 2011(Dhanoa et al., 2015) showed resistance rates of nearly 90% whereas strains from UKMMC from 2010 and 2011 (Biglari et al., 2015) and HSNZ in 2011 (Lean et al., 2014) showed resistance rates of around 70%. Ceftazidime resistance rates for *A. baumannii* isolates from Hospital Selayang in 2010 (Nazmul et al., 2012) were closer to the national resistance rate of 58% for that year, as was the resistance rate for cefepime of isolates from UMMC in 2008–2009 (51%) although the resistance rate for ceftazidime was about 10% higher than the national resistance rate for that period of time (Dhabaan et al., 2012). In stark contrast, all 170 isolates obtained from the ICU of UMMC in 2006–2009 were resistant to ceftazidime and cefepime (Kong et al., 2011). Very high ceftazidime resistance rates had earlier been reported for *Acinetobacter* spp. isolates from UMMC that were isolated in 1987 (81%) and between 1996 and 1998 (97.7%) (Misbah et al., 2004).

The resistance rates for another third generation extended-spectrum cephalosporin, cefotaxime, were consistently higher than ceftazidime and cefepime (**Figure 3**). NSAR first reported the national resistance rates for cefotaxime in 2007 and this was already at 75.4%. An earlier study from HUSM from 2003–2004 showed an even higher cefotaxime resistance rate at 88% (Deris et al., 2009) and this reached 94.7% in strains isolated from the same hospital between 2005 and 2009 (Ariffin et al., 2012). The national resistance rates for cefotaxime remained above 70% for 2009–2012 but dipped slightly below 70% in 2013–2014. Cefotaxime resistance rates for UKMMC in 2010–2011 (Biglari et al., 2015) and HSNZ in 2011 (Lean et al., 2014) were similar to the national resistance rate at that time frame (i.e., around 70%). Interestingly, cefotaxime resistance for *Acinetobacter* spp. isolates from UMMC from 1987 was even higher at 81% and this further increased to 97.7% in isolates obtained from 1996–1998 (Misbah et al., 2004). No data for cefotaxime were available in the NSAR reports for 2015 and 2016.

No NSAR data is also available for the fourth extended-spectrum cephalosporin that was listed in the CLSI and the ECDC-CDC guidelines, i.e., ceftriaxone. However, data from *Acinetobacter* spp. isolates obtained from UMMC in 1987 showed a high resistance rate of 90.5% and this further increased to 97.7% for isolates in 1996–1998 (Misbah et al., 2004). By the following decade, a 100% resistance rate to ceftriaxone was reported for *Acinetobacter* isolates from the UMMC ICU (collected from 2006–2009) (Kong et al., 2011).

### Aminoglycosides

**FIGURE 4:**
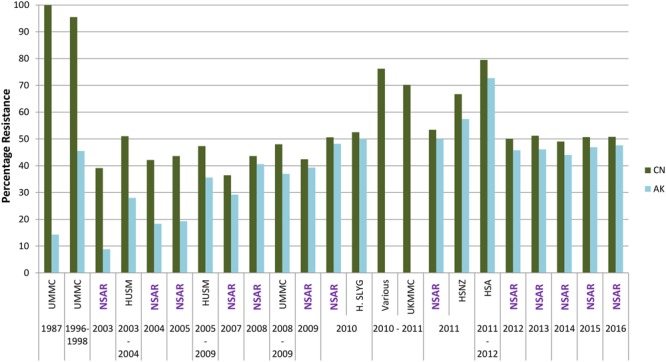
Aminoglycoside resistance rates for Malaysian *Acinetobacter* spp. isolates (1987–2016). CN, gentamicin; AK, amikacin. Data from the National Surveillance for Antibiotic Resistance (NSAR) is included and labeled as “NSAR” in purple-colored fonts. Data from the other studies are as follows: UMMC from 1987 and between 1996 and 1998, (Misbah et al., 2004); HUSM between 2003 and 2006, (Deris et al., 2009); and between 2005 and 2009, (Ariffin et al., 2012); UMMC between 2008 and 2009, (Dhabaan et al., 2012); Hospital Selayang (H. SLYG) in 2010, (Nazmul et al., 2012); UKMMC between 2010 and 2011, (Biglari et al., 2015, 2017); Various, collected from various hospitals mainly around the town of Ipoh in the state of Perak in 2010 and 2011, (Kor et al., 2014); HSNZ in 2011, (Lean et al., 2014); and Hospital Sultanah Aminah (HSA) between 2011 and 2012 (Dhanoa et al., 2015).

The NSAR report from 2003 showed a nationwide gentamicin resistance rate of 39.1% and an amikacin resistance rate that is four-fold lower at 8.8%. Resistance rates steadily increased and by 2008, the resistance rates for both aminoglycosides were similar although the rates for amikacin were around 2–5% lower than that of gentamicin (**Figure 4**). Throughout this period, gentamicin resistance rates increased from 39.1% in 2003 to about 50% in 2010 and remained around that level until the latest NSAR report for 2016. When looking at the aminoglycoside resistance data from individual hospitals as reported by other groups of researchers, the resistance rates for gentamicin were generally higher than for amikacin as shown in the NSAR data (**Figure 4**). However, isolates from three hospitals showed around 20% higher resistance rates than the NSAR data: UKMMC in 2010–2011 (70.2% for gentamicin) (Biglari et al., 2015), HSNZ in 2011 (66.7% for gentamicin, 57.4% for amikacin) (Lean et al., 2014) and HSA in 2011–2012 (79.5% for gentamicin, 72.4% for amikacin) (Dhanoa et al., 2015). A random sample of 42 *A. baumannii* isolates from various hospitals in Malaysia taken from 2008–2009 yielded a gentamicin resistance rate of 76.2% (Kim et al., 2013), which is also above the national resistance rate as reported by NSAR, although for this particular study, the isolates chosen were all carbapenem resistant.

### Fluoroquinolones

Only ciprofloxacin from the fluoroquinolone group of antimicrobials has been used to assess the antimicrobial susceptibility rates for *Acinetobacter* spp. in Malaysia. The NSAR data showed that ciprofloxacin resistance rates increased from about 20% in 2003 to around 50% in 2008 with rates remaining around 50–55% until the latest report for 2016. Results from individual hospitals more or less reflected the national trend with the exception of UKMMC in 2010–2011 which showed a resistance rate of 79.6% (Biglari et al., 2017), HSNZ in 2011 with a rate of 66.1% (Lean et al., 2014) and HSA in 2011–2012 with a rate of 84.1% (Dhanoa et al., 2015). ICU isolates from UMMC (2006–2009) showed highest ciprofloxacin resistance rates at 99.4% (Kong et al., 2011).

### Penicillins

NSAR reported *Acinetobacter* spp. resistance rates for ampicillin and piperacillin from 2007 to 2014. The Malaysian *Acinetobacter* isolates displayed very high resistance rates for ampicillin, which averaged at 89.2% whereas piperacillin showed a lower average resistance rate of 55.6% within the 7-year surveillance period.

### β-Lactam/β-Lactamase Inhibitor Combination

**FIGURE 5:**
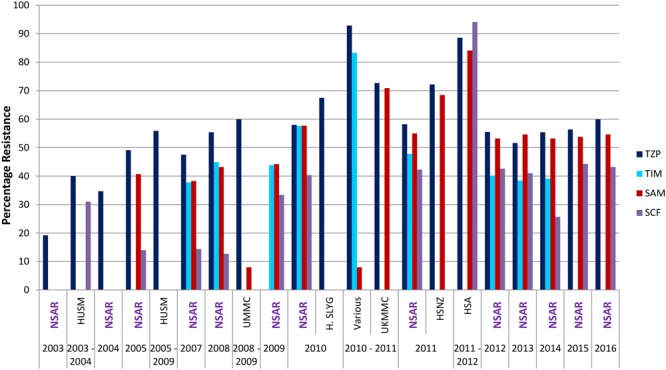
Resistance rates for β-lactam/β-lactamase combination in Malaysian *Acinetobacter* spp. isolates (2003–2016). TZP, piperacillin/tazobactam; TIM, ticarcillin/clavulanate; SAM, ampicillin/sulbactam; SCF, cefoperazone/sulbactam. Data from the National Surveillance for Antibiotic Resistance (NSAR) is included and labeled as “NSAR” in purple-colored fonts. Data from the other studies are as follows: HUSM between 2003 and 2006, (Deris et al., 2009); and between 2005 and 2009, (Ariffin et al., 2012); UMMC between 2008 and 2009, (Dhabaan et al., 2012); Hospital Selayang (H. SLYG) in 2010, (Nazmul et al., 2012); UKMMC between 2010 and 2011, (Biglari et al., 2015, 2017); Various, collected from various hospitals mainly around the town of Ipoh in the state of Perak in 2010 and 2011, (Kor et al., 2014); HSNZ in 2011, (Lean et al., 2014); and Hospital Sultanah Aminah (HSA) between 2011 and 2012 (Dhanoa et al., 2015).

The national resistance rate of *Acinetobacter* spp. toward the combination of piperacillin/tazobactam was relatively low (at 19.2%) in 2003 but this gradually increased to 55.8% by 2008 (**Figure 5**). NSAR data showed that from 2008 to 2016, the national resistance rates for piperacillin/tazobactam remained within the 55–60% range. However, reports of strains isolated from individual hospitals showed markedly higher resistance rates, as had been observed for other antimicrobials. Isolates from HSA in 2011 and 2012 (Dhanoa et al., 2015) showed resistance rates of about 90% whereas the resistance rates were lower at around 70% for UKMMC in 2010 and 2011 (Biglari et al., 2015), and HSNZ in 2011 (Lean et al., 2014) (**Figure 5**).

NSAR data for the combination of ticarcillin/clavulanate was available from 2007–2014 and the national *Acinetobacter* spp. resistance rates remained around the 40% level with the exception of 2010 when it spiked to 57.6% before decreasing to 47.8% the following year (**Figure 5**). The national resistance rate for ampicillin/sulbactam was around 40% from 2005 to 2009, thereafter increasing to between 50 and 60% from 2010 to 2016 (**Figure 5**). Reported resistance rates for the ampicillin/sulbactam combination from individual hospitals were higher, at 84.1% in the HSA *A. baumannii* isolates obtained in 2011 and 2012 (Dhanoa et al., 2015), and about 70% for the UKMMC isolates between 2010 and 2011 (Biglari et al., 2015) and the HSNZ isolates in 2011 (Lean et al., 2014). Lower resistance rates were generally observed for the sulbactam/cefoperazone combination when compared to ampicillin/sulbactam. When NSAR first reported data for sulbactam/cefoperazone in 2005, the resistance rate was at 14% and remained around that level for 2007–2008. The national sulbactam/cefoperazone resistance rate increased considerably to 33.4% in 2009 and it remained between 40 and 45% from 2010 to 2016 with the notable exception of 2014 where the reported rate was at 25.7%. However, *A. baumannii* isolates from HSA (in 2011 and 2012) showed a much higher sulbactam/cefoperazone resistance rate of 94.1%, higher than the ampicillin/sulbactam resistance rate of 84.1% (Dhanoa et al., 2015).

### Tetracyclines

There are very few reports on the prevalence of tetracycline resistance in Malaysian *Acinetobacter* isolates. Lean et al. (2014) reported that out of 54 *A. baumannii* isolates that were collected from various ward in HSNZ in Terengganu during 2011, 87% were resistant to tetracycline while 61.1% were resistant to doxycycline. Similar high resistance rates for tetracycline were reported (79.1%) for a collection of 43 MDR *A. baumannii* isolates that were obtained from various hospitals mainly around the town of Ipoh, Malaysia although the year of their collection and the identity of the hospitals were not stated (Kor et al., 2014).

Tigecycline is a semisynthetic antibiotic belonging to the tetracycline-derived glycylcycline family and along with the lipopeptides or polymyxins (i.e., polymyxin B and colistin, or polymyxin E), tigecycline is considered one of the ‘last resort’ drugs for the treatment of *Acinetobacter* infections (Lim et al., 2011; Doi et al., 2015; Li et al., 2015; Pogue et al., 2015). However, it should be noted that guidelines such as the latest Infectious Diseases Society of America (IDSA) and the American Thoracic Society (ATS) for the management of adults with hospital-acquired pneumonia and ventilator-associated pneumonia (HAP/VAP) strongly recommends against the use of tigecycline in *Acinetobacter* infections (Kalil et al., 2016). Latest systematic reviews and meta-analyses also disfavor the use of a tigecycline-based regimen for the treatment of MDR *A. baumannii* infections, despite its lower nephrotoxicity compared with colistin (Ni et al., 2016; Kengkla et al., 2017). NSAR only reported tigecycline resistance rates for *A. baumannii* blood isolates from 2013–2016 with fairly constant rates of 15–18% for the 4 year period. An earlier study from the UMMC with isolates obtained from 2008–2009 indicated a 5% intermediate susceptibility to tigecycline for their clinical isolates but a much higher percentage (60%) of intermediate susceptibility for hospital environmental isolates (Dhabaan et al., 2012), which is surprising and a cause for concern. On the other hand, Kor et al. (2014) had reported a 58.1% tigecycline resistance rate on their collection of 43 MDR *A. baumannii* from various hospitals in Ipoh but their susceptibility testing for tigecycline was performed using the Kirby-Bauer disk diffusion assay for which no standard breakpoints were available. The 2008–2009 UMMC isolates were assessed for tigecycline susceptibility using both *E*-test and broth microdilution, and the MIC breakpoints from the United States Food and Drug Administration (FDA) were used for their interpretation of tigecycline susceptibility (Dhabaan et al., 2012), a move which was recently supported (Nicolau et al., 2015) in the absence of any CLSI guidelines for tigecycline until now (CLSI, 2017). Broth microdilution is recommended for determining tigecycline MIC values as a report had shown that tigecycline MICs varied greatly according to the *in vitro* testing methods used with Etest giving significantly elevated MICs and were thus, deemed inaccurate (Marchaim et al., 2014).

### Polymyxins (Lipopeptides)

NSAR only reported *A. baumannii* resistance rates for colistin from 2015 onward where rates were low at 0.8% in 2015 and all isolates were susceptible in 2016. Data for the other polymyxin, polymyxin B, was only reported for blood isolates of *A. baumannii* from 2013–2016 with a resistance rate of 1.4% in 2013 and all isolates susceptible in 2014–2016. In stark contrast, Lean et al. (2014) had reported an alarmingly high resistance rate of 25.9% for polymyxin B in HSNZ. So far, this is the only peer-reviewed, published report of polymyxin resistant *A. baumannii* from Malaysia. The UMMC study on *A. baumannii* isolates obtained from 2008 and 2009 did not detect any polymyxin resistance (Dhabaan et al., 2012), as were isolates obtained from the UMMC ICU earlier (between 2006 and 2009) (Kong et al., 2011). Likewise, no polymyxin-resistant isolates were found in the 2011–2012 HSA study (Dhanoa et al., 2015) and the 2010– 2011 UKMMC study (Biglari et al., 2013).

## Resistance Mechanisms

### Carbapenem Resistance

Carbapenem resistance in *Acinetobacter* spp. is now increasingly reported worldwide and is usually mediated by enzymatic inactivation (via carbapenemases), active efflux of drugs and target site modification (i.e., altered penicillin-binding proteins) (Zarrilli et al., 2009). More than 210 β-lactamases belonging to 16 families have been identified in *Acinetobacter* spp. (Zhao and Hu, 2012) with class D β-lactamases being the most widespread carbapenemase in *A. baumannii* (Zarrilli et al., 2009; Bush, 2013). Class B metallo-β-lactamases (MBL; IMP-, VIM-, SIM- and NDM-types) have also been sporadically reported worldwide in *A. baumannii*, being able to hydrolyze carbapenems and other β-lactams, except aztreonam, and resistant to clinically available β-lactamase inhibitors (Zhao and Hu, 2012). Several insertion sequence (IS) elements such as ISAba1, ISAba2, ISAba3 and IS18, have been found to increase the expression of class D β-lactamase genes (including *bla*_OXA-23-like_ and *bla*_OXA-58-like_ genes) when they are inserted immediately upstream due to the presence of an outward-directing promoter at the ends of these IS elements (Zarrilli et al., 2009; Hsu et al., 2017). The *A. baumannnii* chromosome also encodes an intrinsic *bla*_OXA-51-like_ gene that is weakly expressed but does not confer resistance to carbapenems. However, it has been demonstrated that insertion of an ISAba1 element upstream of the gene conferred carbapenem resistance (Turton et al., 2006).

There are very few papers that have investigated the possible carbapenem resistance mechanisms in *Acinetobacter* spp. isolates from Malaysia. The *bla*_OXA-23_ gene appeared to be the predominant acquired carbapenemase in the Malaysian *A. baumannii* isolates, which is not surprising as *bla*_OXA-23_ is the most common cause of carbapenem resistance in *A. baumannii*, and the most widely spread acquired OXA carbapenemase worldwide (Kamolvit et al., 2015). The prevalence of the *bla*_OXA-23_ gene was 75.9% in the 2011 *A. baumannii* HSNZ isolates (Lean et al., 2014) and 82% in the 2010–2011 UKMMC isolates (Biglari et al., 2015, 2017). In an earlier study, nearly 95% of carbapenem-resistant *Acinetobacter* spp. isolated in 2003–2004 from UMMC, were positive for *bla*_OXA-23_ (Wong et al., 2009). However, almost half of the UKMMC isolates that contained the ISAba1-*bla*_OXA-51-like_ configuration were susceptible to carbapenems, leading the authors to conclude that ISAba1 may not upregulate the expression of the intrinsic *bla*_OXA-51-like_ gene and mediate carbapenem resistance (Biglari et al., 2015), as had been previously proposed (Turton et al., 2006). No *bla*_OXA-24-like_ and *bla*_OXA-58-like_ genes were detected so far in the Malaysian *A. baumannii* isolates (Biglari et al., 2015; Lean et al., 2014) although these class D β-lactamases have been found elsewhere, particularly in European isolates (D’Andrea et al., 2009; Merino et al., 2010; Novovic et al., 2015; Chatterjee et al., 2016). Among the Class B MBLs, only *bla*_IMP_ has been reported albeit only in 9.9% of the UKMMC *A. baumannii* isolates (Biglari et al., 2015) and 5.1% in the carbapenem-resistant 2003–2004 UMMC *Acinetobacter* spp. isolates (Wong et al., 2009), whereas neither *bla*_IMP_ nor *bla*_V IM_ was found in the HSNZ *A. baumannii* isolates from 2011 (Lean et al., 2014). Southern hybridization localized the *bla*_IMP-4_ gene in an *A. calcoaceticus* isolate from UMMC to a class 1 integron on an approximately 35 kb plasmid (Wong et al., 2009). Interestingly, genome sequencing of an *A. pittii* isolated in 2014 from a hospital in the state of Perak (in Peninsular Malaysia) led to the discovery of *bla*_NDM-1_ and *bla*_OXA-58_ co-residing in the isolate (Ang et al., 2016). The *bla*_NDM-1_ gene was found within a 10,038 bp composite transposon which resided on a 140 kb megaplasmid whereas the *bla*_OXA-58_ gene was located on a 35 kb plasmid. Metallo-β-lactamase production in this *A. pittii* strain was validated by testing with the Etest MBL kit from BioMériux (Ang et al., 2016).

### Cephalosporin Resistance

*Acinetobacter* spp. are known to encode *Acinetobacter*-specific AmpC cephalosporinases in the chromosome, designated ADCs (Hujer et al., 2005). More than 45 variants of ADCs (ADC-1 to ADC-56) have been categorized for the genus *Acinetobacter* with many more that remain uncategorized (Zhao and Hu, 2012). In cephalosporin-resistant *A. baumannii* isolates from UKMMC, the *bla*_ADC_ gene was present in 93.7% of the isolates and in most of these *bla*_ADC_-positive isolates, ISAba1 was detected upstream of the *bla*_ADC_ gene (Biglari et al., 2015). ADCs are normally expressed at low levels and are not inducible (Hujer et al., 2005) but the insertion of ISAba1 upstream often leads to the overexpression of these cephalosporinases (Héritier et al., 2006). The specific ADC type was, however, not determined for the UKMMC isolates. The *bla*_ADC_ sequence from 3 cephalosporin-resistant *A. baumannii* from HSNZ isolated in 2011 (i.e., AC12, AC29 and AC30) were found to be a hitherto uncategorized ADC (with R80S and G246S mutations in reference to ADC-7) (Lean et al., 2015, 2016). However, these *bla*_ADC_ genes were characterized as belonging to the *ampC* allele 20 in a recent paper reporting on the re-curation of the *A. baumannii*-encoded *ampC* genes in a new database hosted at http://pubmlst.org/abaumannii (Karah et al., 2017). These *bla*_ADC_ genes from *A. baumannii* AC12, AC29 and AC30 were cloned into a pET30a expression vector and expressed in *Escherichia coli* BL21, leading to the recombinant *E. coli* strains displaying resistance to ceftazidime, cefepime, aztreonam and even imipenem (Lean et al., 2016). This suggests that the ADC from these isolates were indeed extended-spectrum *Acinetobacter*-derived AmpC (ESAC) as ADCs typically hydrolyze penicillins, narrow- and extended-spectrum cephalosporins but not carbapenems and zwitterionic cephalosporins such as cefepime (Rodríguez-Martínez et al., 2010; Lean et al., 2016).

### Other Resistance Mechanisms

The main mechanisms of fluroquinolone resistance are mutations that alter the target sites DNA gyrase (encoded by *gyrA* and *gyrB*) and DNA topoisomerase IV (encoded by *parC* and *parE*) (Jacoby, 2005). Ciprofloxacin-resistant *A. baumannii* isolates from UKMMC and *A. baumannii* AC12, AC29 and AC30 from HSNZ all displayed the characteristic serine-to-leucine substitution at position 83 for GyrA and position 80 for ParC (Lean et al., 2015, 2016; Biglari et al., 2017), mutations which have been implicated in fluoroquinolone resistance in *Acinetobacter* (Wisplinghoff et al., 2000; Fournier et al., 2006).

Resistance to polymyxins (polymxin B and colistin) in *A. baumannii* is mediated by multiple factors but is mainly due to modification of the LPS moieties that form the outer membrane layer of the cell (Olaitan et al., 2014; Jeannot et al., 2017; Poirel et al., 2017). In some polymyxin-resistant *A. baumannii*, phosphoethanolamine is enzymatically added to the lipid A of LPS (Arroyo et al., 2011) whereas in other resistant isolates, the LPS part of the outer membrane is completely absent due to mutations in the genes involved in LPS biosynthesis (Moffatt et al., 2010, 2011; Henry et al., 2012). These LPS alterations decrease the net negative charge, preventing the binding of the cationic polymyxin molecules to the bacterial surface (Jeannot et al., 2017; Poirel et al., 2017). PmrAB is a two-component regulatory system that regulates the expression of the genes involved in LPS modification; some mutations in *pmrAB* resulted in polymyxin resistance due to constitutive upregulation of the LPS modification pathway (Arroyo et al., 2011; Park et al., 2011; Lim et al., 2015; Dahdouh et al., 2017). Investigations into the polymyxin-resistant *A. baumannii* isolates from HSNZ in 2011 indicated a P102H mutation in the *pmrA* gene in all resistant isolates and several point mutations in the *lpxC, lpxD* and *lpsB* genes involved in LPS biosynthesis (Lean et al., 2014). Further experimental studies on two of these polymyxin-resistant isolates, *A. baumannii* AC12 and AC30, indicated upregulation of the *pmrB* gene as well as possible impairment (but not total loss) of the LPS (Lean et al., 2016). These mutations are intrinsic, and not transmissible, and are likely the result of selective pressure (Jeannot et al., 2017; Poirel et al., 2017). However, the recent discovery of the transmissible polymyxin-resistant genes, *mcr-1, mcr-1.2*, and *mcr-2* (which encode phosphoethanolamine transferases) in Enterobacteriaceae (Liu et al., 2015; Giamarellou, 2016) raised the alarming possibility of its spread to *Acinetobacter* spp. and other bacteria. Although no reports of *mcr-*positive *Acinetobacter* spp. have emerged until now, it is likely just a matter of time as the *mcr* genes are carried on transmissible plasmids (Malhotra-Kumar et al., 2016; Jeannot et al., 2017). A recent report highlighted this when it was shown that laboratory transformation of an *mcr-1*-encoded recombinant plasmid into several strains of *A. baumannii* led to the development of colistin resistance in these strains (Liu et al., 2017).

## Epidemiology and Genomics

Prior to the current accessibilty of WGS, various molecular methods were available for investigating the epidemiology of *A. baumannii.* Pulsed-field gel electrophoresis (PFGE) was the gold standard for epidemiological investigations of pathogenic bacteria including *A. baumannii* but suffers from limitations such as its labor- and time-intensiveness (2–4 days) and the lack of reliable inter-laboratory reproducibility despite the availability of guidelines for comparison of band positions (Rafei et al., 2014). Other electrophoretic band-based typing methods such as random amplified polymorphic DNA (RAPD) and repetitive sequence-based PCR (Rep-PCR) have been used for *A. baumannii*, but both suffer from lack of intra- and inter-laboratory reproducibility (van Belkum et al., 2007; Rafei et al., 2014). MLST remains the most widely accepted typing technique to study clonality and population structure of *A. baumannii* even in the era of WGS (Zarrilli et al., 2013; Rafei et al., 2014). MLST accesses the genetic variation that occurs in housekeeping genes by considering each unique sequence of the housekeeping gene as an allele type with a sequence type (ST) defined by combination of allele types for each gene in the MLST scheme. There are currently two MLST schemes for *A. baumannii*: (1) the Bartual or the Oxford scheme, which is based on seven genes (*gltA, gyrB, gdhB, recA, cpn60, gpi*, and *rpoD*) (Bartual et al., 2005; Wisplinghoff et al., 2008), and (2) the Institut Pasteur scheme which is also based on seven genes (*cnp60, fusA, gltA, pyrG, recA, rplB* and *rpoB*) (Diancourt et al., 2010), three of which (i.e., *cpn60, recA* and *gltA*) is common with the Oxford scheme.

Despite the availability of various molecular typing methods for *A. baumannii*, papers reporting on the molecular epidemiology of *A. baumannii* in Malaysia are few and far between. *Acinetobacter* isolates from UMMC obtained from 1987 and from 1996–1998 were subjected to Rep-PCR fingerprinting (Misbah et al., 2004) whereas those obtained from the same medical centre in 2006–2009 were analyzed by PFGE (Kong et al., 2011). PFGE profiles revealed the likelihood of a persistent *A. baumannii* clone endemic to the ICU with several environmental isolates and an isolate from the hands of a healthcare worker showing closely related PFGE profiles with isolates from patients (Kong et al., 2011). Similarly, Rep-PCR fingerprints indicated the presence of two distinct *Acinetobacter* lineages at UMMC that could have persisted from 1987 to 1996–1998 (Misbah et al., 2004). However, any meaningful comparisons between these two studies could not be made due to the different fingerprint methods that were used. Hence, an opportunity has been lost to assess the evolution of *Acinetobacter* spp. in the same medical center over a span of two decades. PFGE has also been used to investigate the *A. baumannii* isolates from HSNZ in 2011 (Lean et al., 2014) and *Acinetobacter* spp. isolates from HSA in 2010–2011 (Dhanoa et al., 2015). In both cases, endemicity of a prevalent clone in the respective hospitals as determined by their closely related pulsed-field *Apa*I profiles, was inferred and all isolates belonging to these prevalent clones were carbapenem resistant (Lean et al., 2014; Dhanoa et al., 2015). Clonal relatedness of *A. baumannii* isolates from UKMMC (2010–2011) was assessed by Rep-PCR which indicated 31 clones among the 162 *A. baumannii* isolates at a cutoff value of 90% similarity (Biglari et al., 2017). Unlike the HSNZ and HSA studies, the UKMMC study did not have any strong inference of a prevalent clone within the hospital during the time period of the investigation, based on the Rep-PCR profiles which showed considerable diversity between the isolates (Biglari et al., 2017).

Kim et al. (2013) gave an indication of the Oxford scheme STs that were prevalent in Malaysian *A. baumannii* isolates when they characterized 38 isolates obtained from Malaysia as part of the Asian Network for Surveillance of Resistance Pathogens (ANSORP) study on hospital-acquired pneumonia from 2008–2009. The majority of the Malaysian isolates (30 isolates; 78.9%) belonged to clonal complex 92 (CC92), out of which ST92 (12 isolates; 31.6%), ST195 (7 isolates; 18.4%) and ST426 (7 isolates; 18.4%) were the most frequently identified STs (Kim et al., 2013). Three *A. baumannii* isolates from HSNZ (2011) that were subjected to WGS (namely AC12, AC29 and AC30) were all found to be ST195 (Lean et al., 2015, 2016). Similarly, when MLST was performed on seven selected *A. baumannii* UKMMC isolates (based on their major Rep-PCR profiles), six were found to be ST195 whereas the other isolate was found to be ST208 (Biglari et al., 2017). We mined the GenBank database for *A. baumannii* genome sequences from Malaysia (**Table 2**) and found that only one of the other five available genomes were ST195 (*A. baumannii* strain 461). *A. baumannii* 269 had an unknown ST based on the Oxford scheme but was typed as ST119 using the Pasteur scheme (**Table 1**). Hence, based on the small number of isolates and limited studies that are available, it would appear that the *A. baumannii* isolates from Malaysia mainly belonged to the Global Clone 2 (GC2) CC92, with ST195 being the predominant ST.

**Table 2:** Available whole genome sequences of *A. baumannii* isolated from Malaysia in the NCBI GenBank database.

*A. baumannii* strain	Source of isolate	ST (Oxford scheme)	ST (Pasteur scheme)	Accession number	Reference^∗^
AC12	Blood	ST195	ST2	CP007549.3	Lean et al., 2015
AC29	Endotracheal secretion	ST195	ST2	CP007535.2	Lean et al., 2016
AC30	Endotracheal secretion	ST195	ST2	CP007577.1	Lean et al., 2016
PR07	Blood	ST734	ST239	CP012035.1	Izwan et al., 2015
269	Mucoid sputum	Unknown	ST119	JQNV00000000	NA
863	Mucoid sputum	ST938	ST2	LZTF00000000	NA
461	Wound swab	ST195	ST2	LCTE00000000	NA
341	Mucopurulent sputum	ST938	ST2	JQSD00000000	NA

*^∗^NA, not available.*

## Conclusion

In this review, we have comprehensively examined the trends of antimicrobial resistance in *Acinetobacter* spp. isolated from various hospitals in Malaysia covering a period of nearly three decades from 1987 to 2016. The national *Acinetobacter* spp. carbapenem resistance rate currently stands at around 60%, which is similar to the levels reported for 2015 in two of Malaysia’s neighboring countries which have national surveillance programs, i.e., Singapore (50%), and the Philippines (54.1%), whereas Thailand reported a higher rate of 73.7% (Hsu et al., 2017). The major acquired carbapenemase gene in *Acinetobacter* spp. isolated from Malaysia is *bla*_OXA-23_, as had been reported in these three neighboring countries although it should be noted that these data were obtained from individual studies and not through their respective national surveillance programs (Hsu et al., 2017). Although results from the Malaysian national surveillance program, NSAR, are publically available online from 2003 onward, the data and analysis could be vastly improved, as we had pointed out here and in a recent commentary (McNeil et al., 2016). Good quality surveillance data is an important component in the global fight against the spread of antimicrobial resistance and the paucity of such essential epidemiological data often leads to delayed or suboptimal revisions in policies and guidelines, which in turn, strengthens the vicious cycle of the careless use of antibiotics by medical practitioners (Laxminarayan et al., 2013). Ideally, a comprehensive surveillance programme should also include molecular epidemiological testing which would enable us to have an in-depth understanding of the origins and extent of the antimicrobial resistance problem (Hsu et al., 2017) but this will likely not be implemented in the near future due to the limited resources of these countries with the exception of perhaps Singapore. Closer collaborations between institutes that handle the national surveillance programs with other academic or research institutions with the relevant resources and skills for molecular epidemiology and WGS should be fostered to better expedite and improve the quality of the surveillance data. This is particularly pressing for priority pathogens such as *Acinetobacter* spp. for which containing and preventing the spread of antimicrobial resistance is of paramount importance to prevent a possible “antibiotic apocalypse” whereby such bacterial infections would no longer be treatable with antibiotics.

## Author Contributions

Conception and design of study: CCY, NIAR, SI, and SCC; acquisition of data: FMR and AGA; analysis and interpretation of data: FMR, CCY, AGA, DWC, and SCC; drafting of the manuscript: FMR and CCY; critical revisions of the manuscript: NIAR, SI, AGA, DWC, and SCC. All authors have approved the final article.

## Conflict of Interest Statement

The authors declare that the research was conducted in the absence of any commercial or financial relationships that could be construed as a potential conflict of interest.

## Footnotes

1 http://www.imr.gov.my/en/component/content/article/75-english-content/national-collabration/1469-nsar.html

## Abbreviations

*Abc* complex: *Acinetobacter baumannii–calcoaceticus* complex
ADC: *Acinetobacter*-derived cephalosporinase
CC: clonal complex
HSA: Hospital Sultanah Aminah
HSNZ: Hospital Sultanah Nur Zahirah
HUSM: Hospital Universiti Sains Malaysia
IMR: Institute of Medical Research
LPS: lipopolysaccharide
MBL: metallo-β-lactamase
MDR: multidrug resistance
MLST: multilocus sequence typing
NSAR: National Surveillance of Antibiotic Resistance
UKMMC: Universiti Kebangsaan Malaysia Medical Centre
UMMC: University of Malaya Medical Centre
WGS: whole genome sequencing
